# Differential responses of heterotrophic and autotrophic respiration to nitrogen addition and precipitation changes in a Tibetan alpine steppe

**DOI:** 10.1038/s41598-018-34969-5

**Published:** 2018-11-08

**Authors:** Changbin Li, Yunfeng Peng, Xiuqing Nie, Yuanhe Yang, Lucun Yang, Fei Li, Kai Fang, Yuanming Xiao, Guoying Zhou

**Affiliations:** 1 Key Laboratory of Tibetan Medicine Research, Northwest Institute of Plateau Biology, Chinese Academy of Science, Xining, 810008 China; 2 Qinghai Key Laboratory of Qing-Tibet Biological Resources, Xining, 810008 China; 3 State Key Laboratory of Vegetation and Environmental Change, Institute of Botany, Chinese Academy of Sciences, Beijing, 100093 China; 4 University of Chinese Academy of Science, Beijing, 100049 China

## Abstract

Soil respiration (Rs) is an important source of atmospheric CO_2_ flux and is sensitive to changes in soil nutrient and water contents. Despite extensive studies on the effects of enhanced atmospheric nitrogen (N) deposition and changes in precipitation (P) on Rs, few studies have taken into account the effects of interactions between these factors on Rs of alpine grasslands. To address these questions, we investigated the effects of N addition (10 g N m^−2^ yr^−1^), changes in precipitation (±50% precipitation), and their interaction on soil respiration and its components, including heterotrophic respiration (Rh) and autotrophic respiration (Ra),in a Tibetan alpine steppe during three consecutive growing seasons. We found that Rs differed in its response to N addition and precipitation regimes. Specifically, decreased precipitation led to a significant reduction in Rs during the last two years, whereas N addition minimally impacted Rs. Another important finding was that soil respiration components differed in their response to N addition and precipitation regimes. Nitrogen addition significantly enhanced Ra, whereas Rh was not altered in response to N addition. By contrast, the precipitation regime led to marked changes in Rh, but exhibited marginally significant effects on Ra. Therefore, our findings highlighted that soil respiration differed in its response to N addition and precipitation regimes mainly due to the different responses of soil respiration components to these factors. Therefore, carbon dynamics should take soil respiration components into account under global change scenarios.

## Introduction

As the second-largest carbon (C) flux, soil respiration (Rs) plays a critical role in the regulation of C cycling in terrestrial ecosystems. Researchers have estimated that Rs accounts for 77 petagrams (Pg) of C released to the atmosphere each year^1,2^. Because grasslands account for approximately 37% of the world’s terrestrial region^2^, C releases from grassland soils significantly contribute to the global carbon cycle. Rs is composed of autotrophic respiration (Ra), which is related to roots, mycorrhizae and other rhizospheric microorganisms, and heterotrophic respiration (Rh) from non-rhizospheric microorganisms and free-living soil^3–5^. In the context of global changes, the response of Rsis mainly mediated by Ra and Rh related to biotic and abiotic factors directly or indirectly^6,7^. However, in recent years, as the importance of monitoring Ra and Rh been increasingly recognized, more and more experiments have started to measure Ra and Rh^8,9^. Numerous biotic and abiotic factors are assumed to regulate Rs and its components. N addition will likely affect Rs via impacts on these drivers^10^. Consequently, Ra and Rh are indispensable for evaluating the carbon balance of terrestrial ecosystems. However, we know little about how these factors directly and indirectly regulate Ra and Rh in the context of N addition.

Nitrogen (N) availability regulates global carbon balance^11^. Over recent decades, the burning of fossil fuel and use of fertilizer have increased the input of N into terrestrial ecosystems^12,13^, and this trend will continue to increase in the future^14^. The effects of N addition on Rs have been reported in different ecosystems, such as forest ecosystems^15–17^, temperate grasslands^18–21^ and other ecosystems. Nevertheless, the results are disputable and may be positive^22,23^, negative^16^ or neutral^15,24,25^. The reason for these differences may result from varied experimental duration and N addition rates. For instance, Ren found that N addition increased the Rs at an earlier stage but reduced Rs in a later period^6^. The effects of N availability on Rs were generally evaluated in previous studies, but the responses of these processes on the interaction of N additions and precipitation changes remain limited, particularly in this semi-arid grassland.

Compared with numerous studies examining the effects of N on Rs, our understanding of precipitation changes on Rs and its components is very limited, and information from the Qinghai-Tibetan Plateau is particularly lacking. More extreme precipitation events and drought are predicted to occur in the future^26^, and such changes will likely significantly affect the soil moisture and thus Rs. Nevertheless, compared with drought, few studies have evaluated the influence of heavy precipitation on Rs^27–29^. To date, the number of precipitation manipulation studies separating Rs components is very limited^30^. In addition, these studies simulated increased rainfall amounts through water addition, which may not necessarily reflect the real conditions of natural extreme rainfall events that may exceed past and current variation ranges^31–33^. Knapp developed a conceptual model called the Bucket Model to predict the response of terrestrial ecosystems to increased intra-annual precipitation variability characterized by extreme precipitation events and longer dry intervals^29^. The Bucket Model predicts that the primary productivity and Rs in arid lands is increased by the increased precipitation variability^34^. To date, it remains unknown how precipitation changes affect soil respiration in cold and dry environments, such as the Tibetan alpine steppe.

As the largest and highest plateau in the world, Qinghai-Tibetan Plateau has a mean elevation of greater than 4000 m and an area of 2.0 × 10^6^ km^2,35^ and is one of the most sensitive areas to climate change. As the dominant ecosystems on the plateau, alpine steppe and alpine meadow occupied greater than 60% of the total region. Atmospheric N deposition is significant in the eastern Plateau due to regional economic development, ranging from 8.7 to 13.8 kg N ha^−1^ yr^−1^ ^36^. Unlike N deposition, the precipitation pattern varied across the Qinghai-Tibetan Plateau, which increased in the northern area and decreased in the southern area^37^. Nevertheless, few studies have concentrated on the influence of precipitation changes and N addition on Rs in alpine steppe.

In this study, we conducted a field precipitation and N manipulative experiment in an alpine steppe, and we aimed to investigate the responses of soil respiration and its components to precipitation changes and N additions. Our objectives were to (i) reveal the response patterns of Rs and its constituents to N addition and precipitation changes, and (ii) evaluate the impacts of biotic and abiotic variables on Ra and Rh through N addition and precipitation changes.

## Results

### Seasonal variations in Microclimates

**Figure 1:**
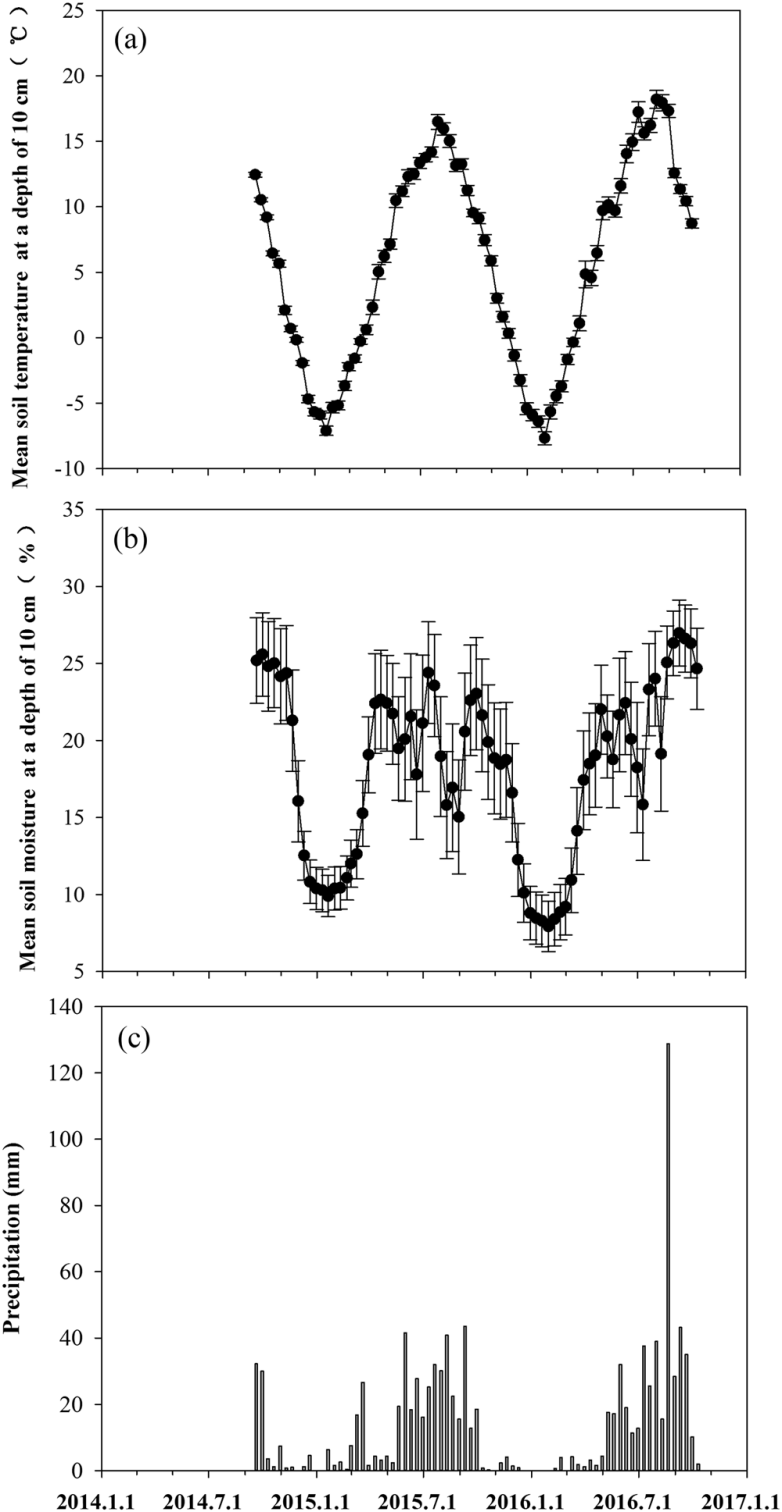
Seasonal and annual variation in (**a**) soil temperature at a depth of 5 cm; (**b**) volumetric soil moisture at a 5-cm depth; (**c**) precipitation from May in 2014 to October in 2016. Error bars indicate the standard errors.

Figure 1 presents the seasonal patterns of soil temperature, soil water content and precipitation from September 2014 to October 2016. The ten-day mean soil temperature ranged from −7.67 to 18.20 °C at a 0- to 10-cm soil depth (Fig. 1a), and mean soil moisture ranged from 7.93 to 26.97 V/V% at a 0- to 10-cm soil depth (Fig. 1b). The 10-day precipitation ranged from 0 to 128.8 mm. During the growing season, the total precipitation was 502.5 mm in 2014, 367 mm in 2015 and 473.4 mm in 2016 (Fig. 1c).

### Soil temperature and moisture

**Figure 2:**
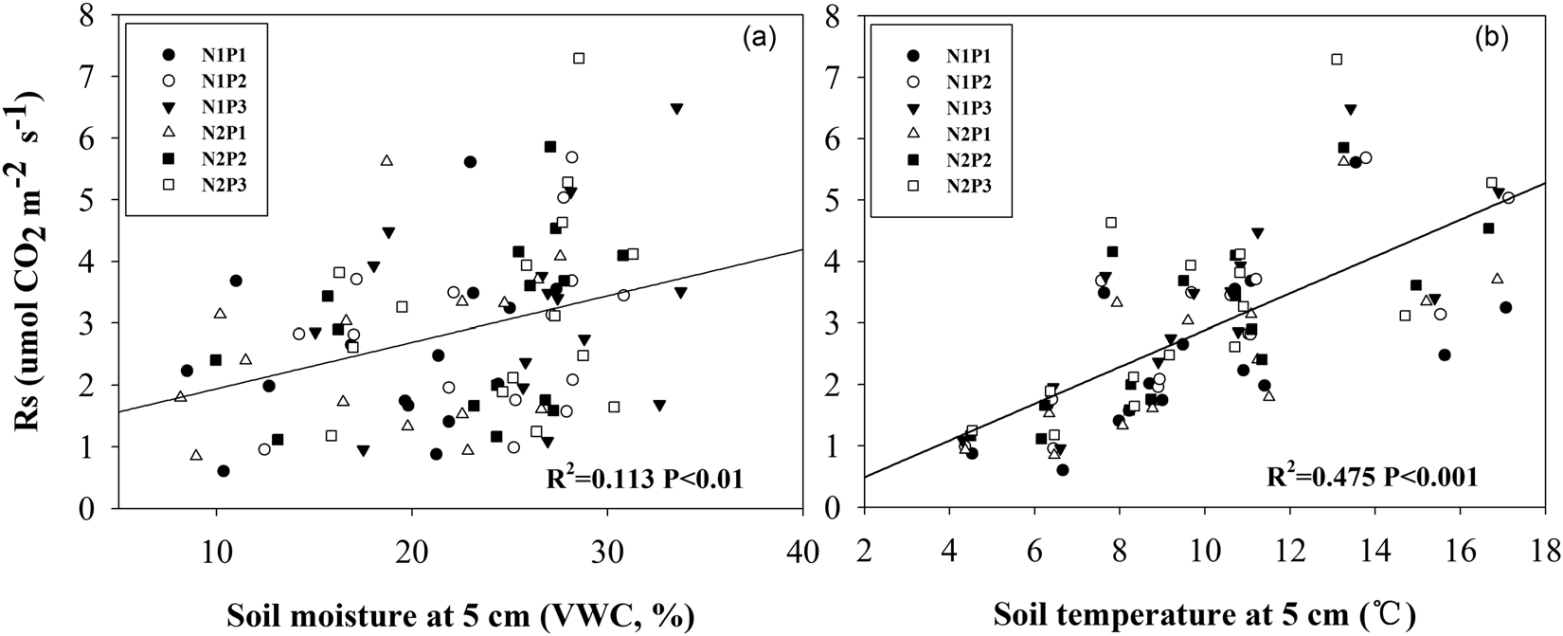
Relationships of Rs with seasonal mean soil temperature and moisture across the 30 plots. Black solid lines represent regression curves.

Given control of soil moisture and soil temperature over soil respiration, we examined the dynamic changes in soil temperature and soil moisture in the growing season. Our results demonstrated that N addition significantly reduced soil temperature rather than soil moisture (Table 1). By contrast, increased precipitation led to a significant increase in soil moisture, whereas reduced precipitation significantly reduced soil moisture (Table 1). However, precipitation did not affect soil temperature (Table 1). Moreover, we found that N- and precipitation-induced changes in soil respiration were closely associated with soil temperature and moisture (Fig. 2).

**Table 1:** F-ratios of the effects of nitrogen (N), precipitation (P), measuring date (D) and their interaction on soil temperature and moisture at a 10-cm depth from 2014 to 2016.

Variable	Soil temperature at a 10-cm depth	Soil moisture at a 10-cm depth
Date (D)	1272.418***	467.103***
Nitrogen (N)	20.058***	0.372^ns^
Precipitation (P)	1.734^ns^	10.757**
D × N	2.038***	1.388*
D × P	1.823***	6.278***
N × P	1.106^ns^	0.988^ns^
D × N × P	0.921^ns^	2.496***

***P < 0.001, **P < 0.01, *P < 0.05; ns indicates no significance.

### Effects of N addition and precipitation change on soil nitrogen availability

Consecutive N addition significantly enhanced soil N availability. For example, NH_4_^+^-N and NO_3_-N concentrations in soil were enhanced by 49.01% and 164.89%, respectively. However, NH_4_^+^-N and NO_3_-N concentrations in soil exhibited differential responses to precipitation. Specifically, precipitation had a significant effect on NO_3_-N concentrations in soil but no impact on NH_4_^+^-N concentrations in soil (Table 2). For example, decreased precipitation enhanced soil NO_3_-N concentrations by 38.01%, whereas enhanced precipitation reduced NO_3_-N concentrations by 20.16% (Table 2). Moreover, the stimulatory effects of precipitation on NO_3_-N concentration were much stronger in 2015 compared with 2016 (Table 2).

**Table 2:** Summary of three-way analysis of variance (ANOVA) of nitrogen (N), precipitation (P) and year (Y) on soil NH_4_^+^-N and NO_3_^_^Nat a 10-cm depth from 2014 to 2016.

Variable	Soil NH_4_^+^-N at a 10-cm depth	Soil NO_3_^−^N at a 10-cm depth
Year (Y)	12.382***	5.645***
Nitrogen (N)	29.067***	79.114***
Precipitation (P)	0.945^ns^	10.036***
Y × N	3.555*	9.104***
Y × P	0.438^ns^	0.749^ns^
N × P	3.086*	6.806**
Y × N × P	0.298^ns^	2.500*

***P < 0.001, **P < 0.01, *P < 0.05; ns indicates no significance.

### Effects of N addition and precipitation change on aboveground and below-ground biomass

**Figure 3:**
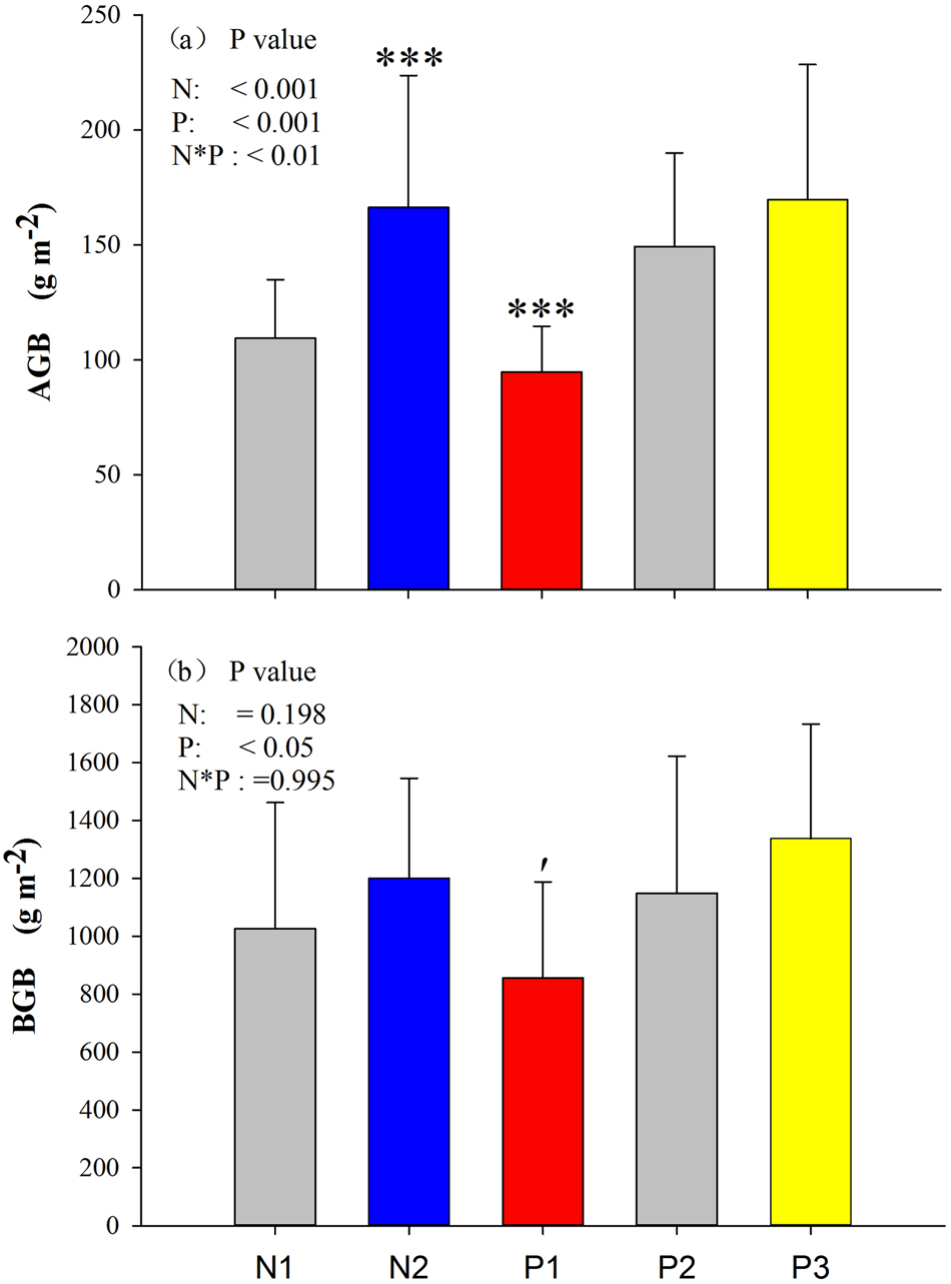
Effects of nitrogen and precipitation changes on (**a**) aboveground biomass (AGB), (**b**) belowground biomass (BGB) in 2016. N1 indicates CK, nitrogen addition (N2), 50% reduction in precipitation (P1), natural precipitation (P2), 50% additional precipitation (P3), ′*P* < 0.1, *P < 0.05, ***P < 0.001.Error bars indicate the standard errors.

Above-ground biomass (AGB) of the community was significantly enhanced by N addition, whereas N addition had no effect on below-ground biomass (BGB). Similar to N addition, decreased precipitation significantly reduced AGB. However, decreased precipitation marginally reduced BGB, whereas enhanced precipitation had no effect on BGB (Fig. 3).

### Soil respiration and its Constitute during the growing season

**Figure 4:**
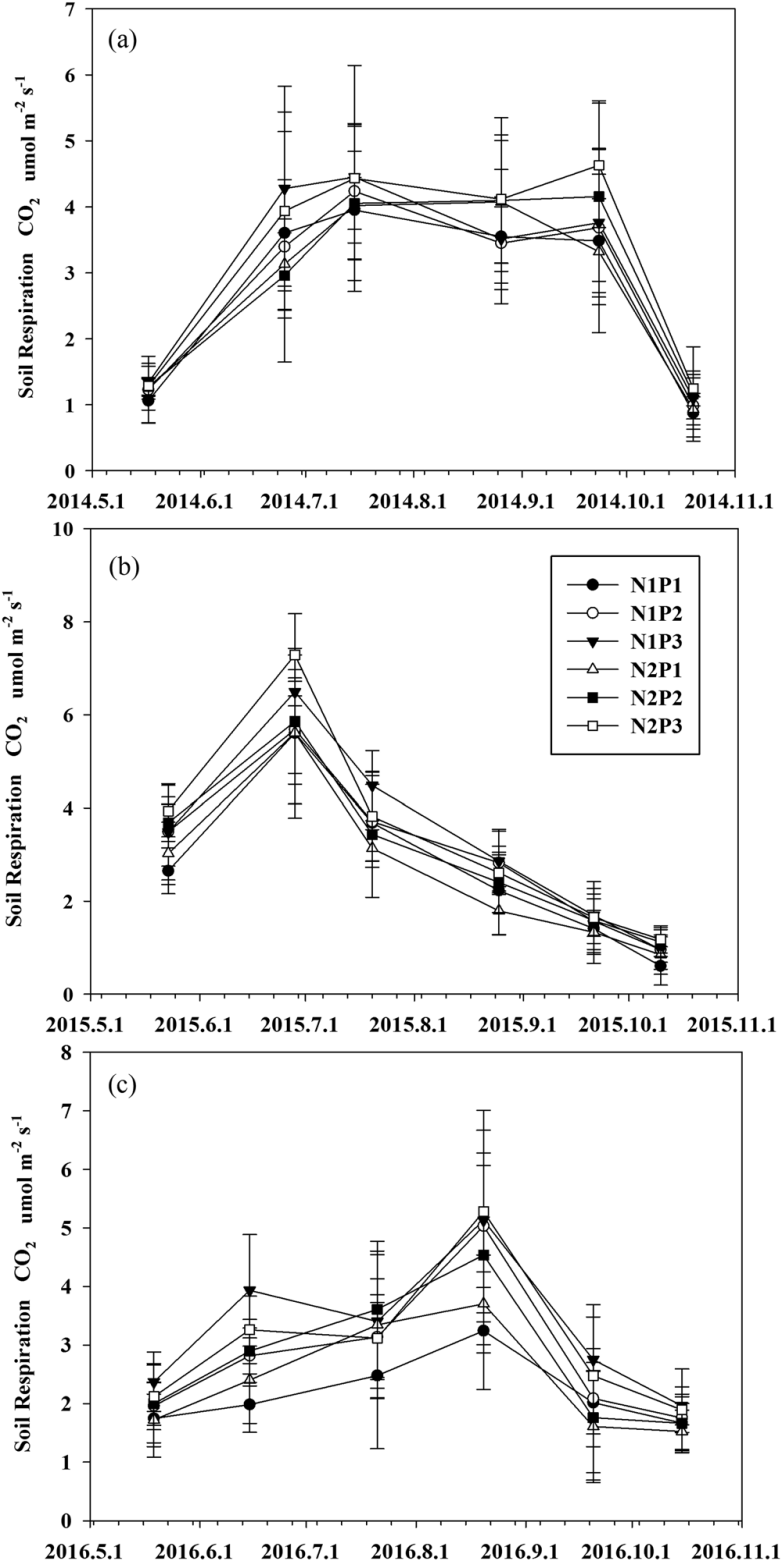
Seasonal variation in soil respiration rates under different treatments in (**a**) 2014; (**b**) 2015; (**c**) 2016. N1P1 indicates the 50% precipitation reduction treatment, natural precipitation (N1P2), 50% additional precipitation treatment (N1P3), 50% reduction in precipitation with nitrogen addition treatment (N2P1), natural precipitation without nitrogen addition (N2P2), 50% additional precipitation with nitrogen addition (N2P3). Error bars indicate the standard errors.

**Figure 5:**
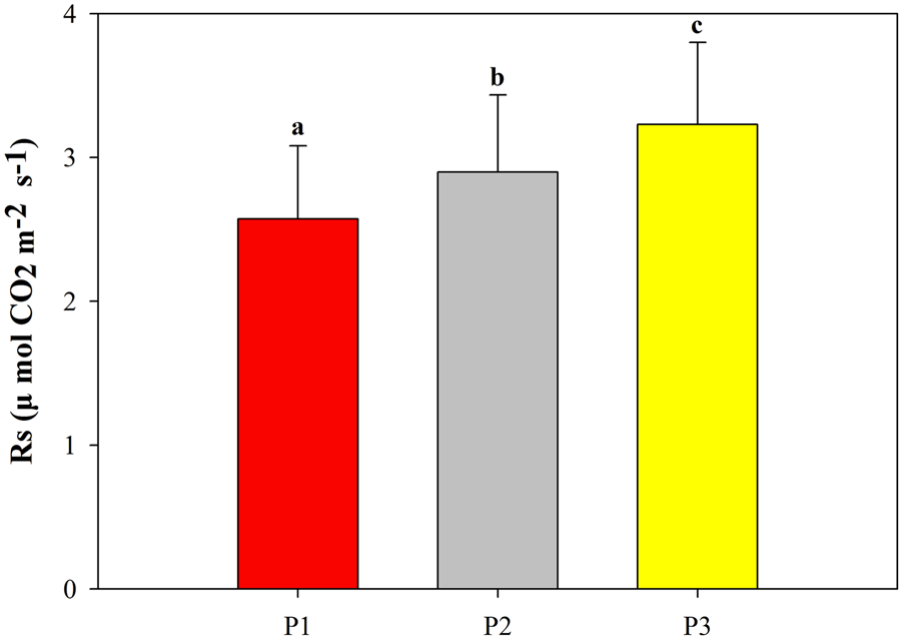
Effects of precipitation changes on mean soil respiration during the growing seasons from 2014 to 2016. P1 indicates 50% reduction in precipitation, natural precipitation (P2), 50% additional precipitation (P3). Error bars indicate the standard errors.

The soil respiration rate exhibited a unimodal pattern in the growing season. The pattern was consistent in the consecutive 3-year observations from different treatments (Fig. 4). For example, soil respiration peaked in July to August and subsequently declined thereafter (Fig. 4). Notably, the peak of soil respiration in 2015 was much greater than that in 2014 and 2016 (Fig. 4). Moreover, N addition and precipitation exhibited differential effects on soil respiration. For example, consecutive N addition had no effects on soil respiration (Fig. 5). By contrast, increased precipitation significantly increased soil respiration, where as reduced precipitation led to a significant reduction in soil respiration (Fig. 5).

**Figure 6:**
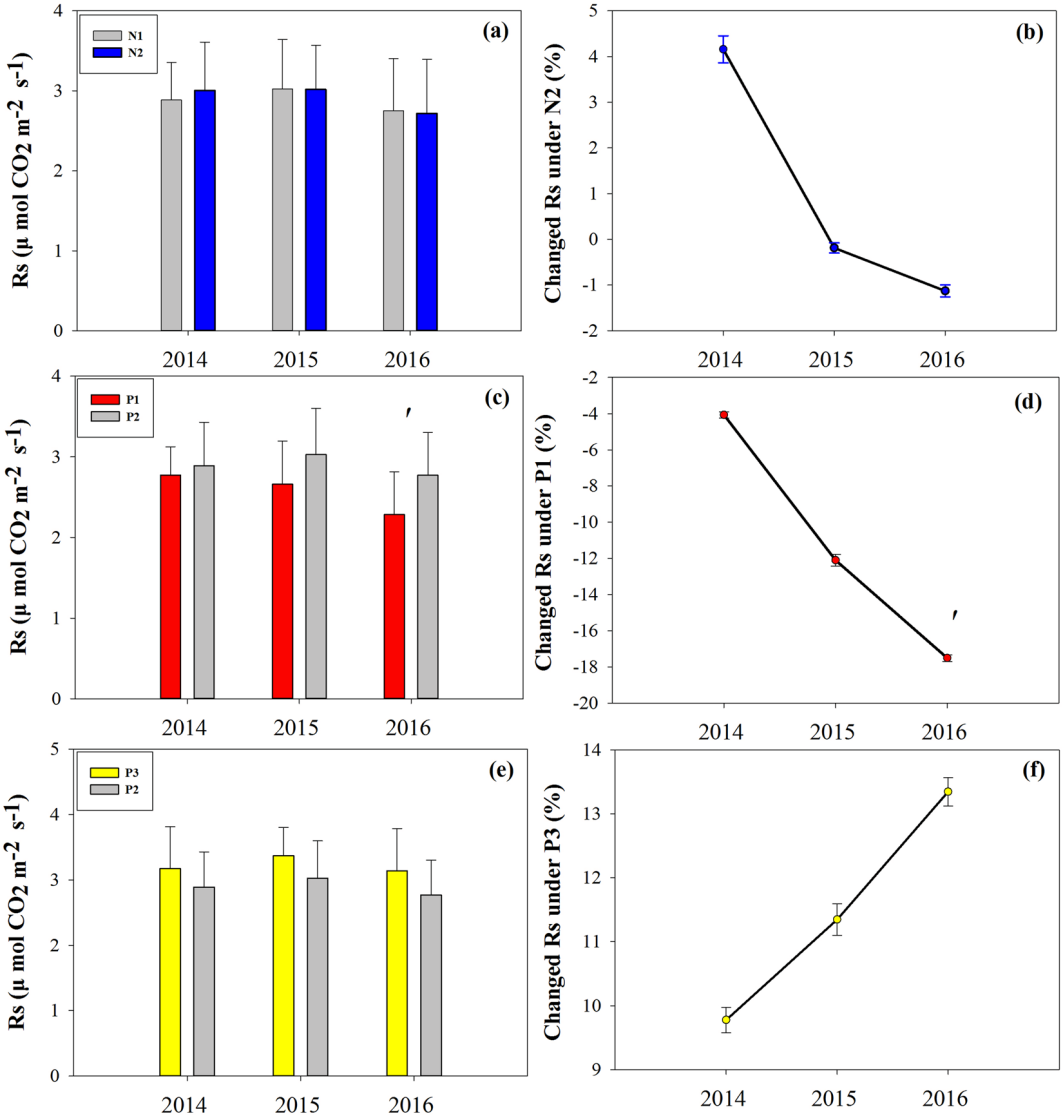
Effects of precipitation changes and N addition on soil respiration (**a**,**c**,**e**) and changes (%) in soil respiration (**b**,**d**,**f**) during the growing seasons from 2014 to 2016. N1 indicates CK, nitrogen addition (N2), 50% reduction in precipitation (P1), natural precipitation (P2), 50% additional precipitation (P3), ^**′**^*P* < 0.1, *P < 0.05, ***P < 0.001, Error bars indicate the standard errors.

In the growing seasons, precipitation changes and N addition were observed from 2014 to 2016. N addition (N2) enhanced Rs by 4.16% in 2014 and reduced Rs by 0.19% and 1.13% in 2015 and 2016, respectively; however, these effects were not significant (Table 3; Fig. 6a,b). Similarly, the effect of P3 treatment on Rs was significant (Table 3; Fig. 6e,f). Compared with the P2 treatments, P1 treatments significantly decreased Rs by 17.5% (*P* = 0.082) in 2016, and in no significant effects were noted in 2014 and 2015 (Table 3; Fig. 6c,d).

**Table 3:** F-ratios of the effects of nitrogen (N), precipitation (P), measuring date (D) and their interaction on soil respiration (Rs).

Variables	2014	2015	2016
Date (D)	115.261***	274.156***	69.038***
Nitrogen (N)	0.353^ns^	0.001^ns^	0.020^ns^
Precipitation (P)	1.392^ns^	4.227*	5.157*
D × N	1.783^ns^	2.612*	0.834
D × P	0.878^ns^	1.708^ns^	2.414*
N × P	0.558^ns^	0.978^ns^	0.889^ns^
N × D × P	0.269^ns^	0.281^ns^	0.711^ns^

***P < 0.001, **P < 0.01, *P < 0.05; ns indicates no significance.

**Figure 7:**
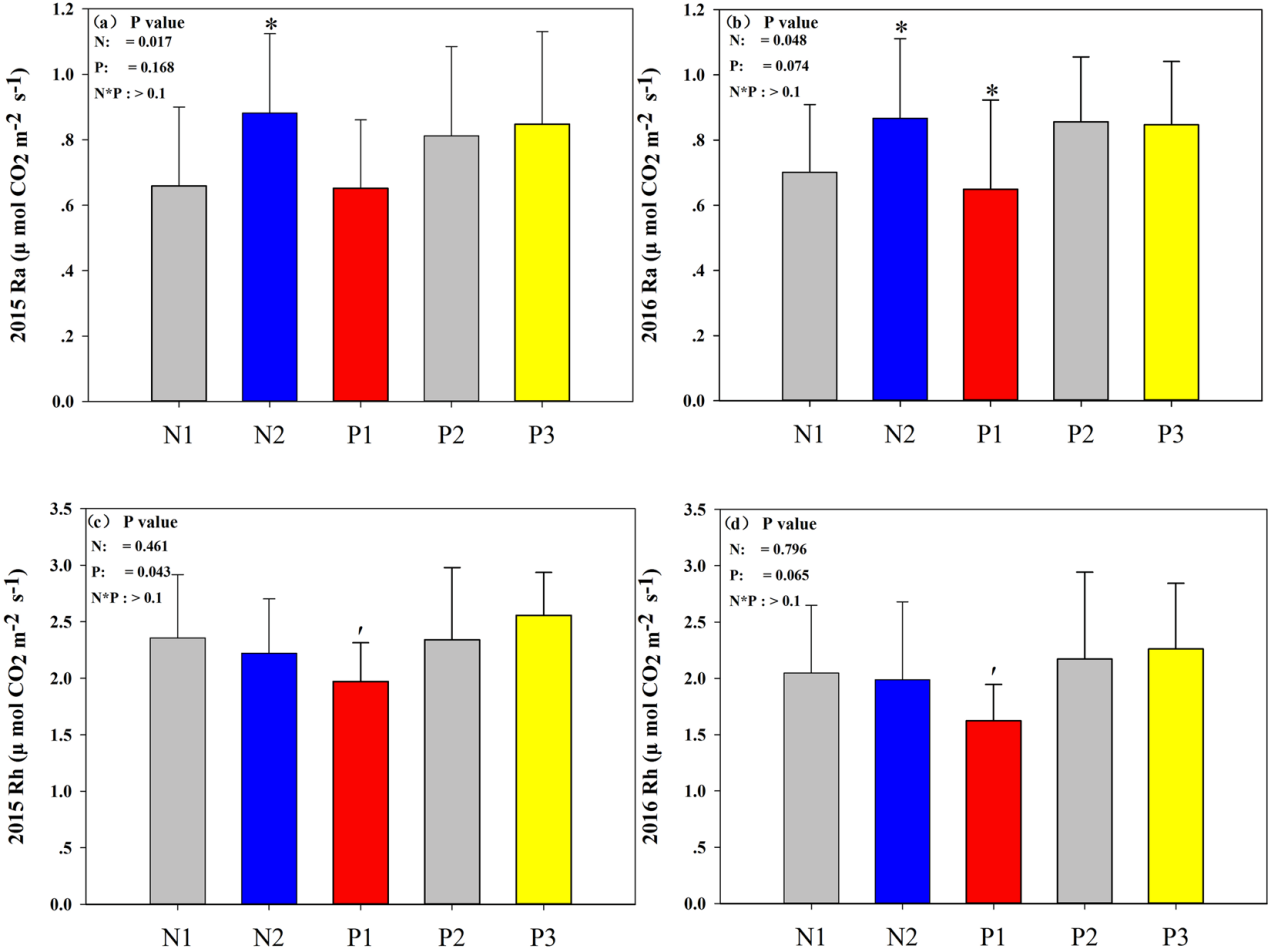
Effects of nitrogen addition and precipitation changes on (**a**) autotrophic respiration (Ra) in 2015, (**b**) autotrophic respiration (Ra) in 2016, (**c**) heterotrophic respiration (Rh) in 2015 and (**d**) heterotrophic respiration (Rh) in 2016. N1 indicates CK, nitrogen addition (N2), 50% precipitation reduction (P1), natural precipitation (P2), 50% additional precipitation (P3), ′*P* < 0.1, *P < 0.05, *** *P* < 0.001. Error bars indicate the standard errors.

To further investigate the effects of N addition and precipitation on soil respiration components, we monitored autotrophic and heterotrophic respiration. Our results demonstrated that the two types of soil respiration differed in their responses to N addition. Specifically, N addition significantly enhanced autotrophic respiration but had no effects on heterotrophic respiration (Fig. 7a,b; Table 4). Moreover, despite the lack of response of autotrophic respiration to precipitation, decreased precipitation led to a significant reduction in heterotrophic respiration. In particular, in 2016, decreased precipitation had minimal impacts on autotrophic respiration (Fig. 7c,d; Table 4). In addition, only the contribution of Rh to the total Rs was reduced by N2 (Rh/Rs, by 6.85%, Table 5).

**Table 4:** F-ratios of the effects of nitrogen (N), precipitation (P), measuring date (D) and their interaction on Rh and Ra.

Variable	2015 Rh	2016 Rh	2015 Ra	2016 Ra
Date (D)	214.728***	75.184***	16.350***	5.204***
Nitrogen (N)	0.777^ns^	1.116^ns^	6.535*	4.278*
Precipitation(P)	3.557*	4.191*	1.973^ns^	2.844^ns^
D × N	1.309^ns^	2.615*	0.814^ns^	3.420**
D × P	3.403**	2.030*	0.842^ns^	0.819^ns^
N × P	0.138^ns^	0.140^ns^	0.474^ns^	0.409^ns^
N × D × P	0.508^ns^	0.663^ns^	0.260^ns^	0.715^ns^

***P < 0.001, **P < 0.01, *P < 0.05; ns indicates no significance.

**Table 5:** F-ratios of the effects of nitrogen (N), precipitation (P), measuring date (D) and their interaction on Ra/Rs.

Variable	2015Rh/Rs	2016 Rh/Rs	2015Ra/Rs	2016 Ra/Rs
Date (D)	2.792*	5.100***	2.792*	5.100***
Nitrogen (N)	**3.820′**	2.496^ns^	**3.820′**	2.496^ns^
Precipitation(P)	1.108^ns^	0.137^ns^	1.108^ns^	0.137^ns^
D × N	0.291^ns^	2.551*	0.291^ns^	2.551*
D × P	2.176*	1.195^ns^	2.176*	1.195^ns^
N × P	0.591^ns^	0.466^ns^	0.591^ns^	0.466^ns^
N × D × P	0.403^ns^	0.550^ns^	0.403^ns^	0.550^ns^

***P < 0.001, **P < 0.01, *P < 0.05, ′*P* < 0.1; ns indicates no significance.

## Discussion

In the present study, we found that the addition of and altered precipitation had differential effects on soil respiration in a Tibetan alpine steppe plateau (Fig. 6). Soil respiration in the growing season exhibited no response to consecutive N addition for three years (Fig. 6a,b). By contrast, increased precipitation led to a significant increase in soil respiration, whereas reduced precipitation significantly reduced soil respiration (Fig. 6 and Table 3), suggesting that changes in soil respiration in this ecosystem might be driven by precipitation limitations.

### Effects of N addition

A recent meta-analysis demonstrated that N addition, increased Rs and Ra but reduced Rh in grasslands^22^. However, we found that the response of soil respiration to N addition was not significant (Table 3; Fig. 6a,b), and these results were similar to previous studies^24,38,39^. Although the effects of N addition are not significant, a slight trend is observed in the first year wherein the addition of N increased Rs. However, Rs values during the subsequent two years were not significantly altered or decreased (Fig. 4a,b). Similar temporal responses were observed in an alpine meadow, in which N addition increased Rs during the first year, had no effect during the following two years, and decreased Rs rates in the last year^6,40,41^. This finding suggests that the response of soil respiration to N addition is strongly dependent on the duration of N addition. These results are consistent with recent investigations in the same region^10^. Of note, our study only presented the responses of Rs to short-term N additions (three years), but it remains undetermined whether long-term N enrichment would yield some differences. Therefore, experiments with a longer duration are required to better understand Rs and Ra/Rh relationships in future studies.

Rs was not altered by N addition (Fig. 6a,b). However, another important finding is that Ra was significantly increased by N addition (Table 4). The Ra response was similar to the response of aboveground biomass and belowground biomass (Fig. 2). Belowground biomass had a direct effect on Ra because larger root biomass represents a larger root surface area for respiration^10,22^. Similar responses to N addition have been observed in a meta-analysis by Xia & Wan^42^. The meta-analysis demonstrated that total root biomass increased by 23%, suggesting that N addition increased biomass. Greater root biomass indicates that more C was allocated below ground^43^, which is typically associated with increased root respiration^44^. However, upon continual nitrogen input into alpine steppe ecosystems, nitrogen saturation will ultimately be achieved, which will cause the root biomass to plateau^10,22^. We observed that N addition did not significant increase BGB in 2016. However, a recent meta-analysis found that the addition of N significantly increased BGB on the Tibetan Plateau^45^. Although the BGB was not significantly altered, we observed an increasing trend for N addition in 2016 in this study (17.01%; Fig. 2a,b). The different findings may be attributed to different soil temperature (Table 1), the soil temperature in our experiment was significantly decreased by N addition. For vegetation growth, temperature is a limiting factor in the alpine steppe on the Tibetan Plateau, and thus higher temperature may stimulate vegetation productivity^46^. Moreover, the soil’s inorganic N concentration increased with the addition of N (Table 2). The high inorganic N accumulated in 0–10 cm of soil may be toxic to microbial activities and growth^47^.

A previous study in an alpine steppe indicated that both plant growth and microbial activity were limited by N^48^. However, long-term N input to N-limited soils should initially stimulate soil microbial activity and lead to carbon-limited conditions after the microbial requirements for N are achieved^47^. This limitation of C to microorganisms under conditions of supplementation with N would lead to reductions in Rh and inhibition of microbial activity^25,49^. Furthermore, we observed that soil temperature exhibited a decreasing trend with increasing N additions (Table 1). Soil temperature not only directly explained the variations in Ra and Rh by adjusting respiration enzymes^50^ but also indirectly influenced these values via affecting microbial and root growth^16^. These effects were particularly noted in cold areas where temperature is generally considered a limiting factor for living things^51^. However, a recent meta-analysis found that N addition did not significantly affect soil temperature on the Tibetan Plateau^45^. This finding is potentially explained by the fact that the addition of N increased aboveground plant growth, and a more closed canopy prevented the incoming solar radiation from reaching the soil surface^52^. Moreover, we observed a significant correlation between soil respiration and soil moisture/temperature (Fig. 2). To our knowledge, soil moisture is mainly mediated by soil temperature^53^. For example, increased soil temperature often reduces soil moisture^53^. However, N-induced reductions in soil temperature rarely reduce soil moisture. Therefore, soil respiration in the Tibetan alpine steppe plateau was regulated by soil moisture. This may represent an important mechanism by which N addition and altered precipitation affect soil respiration.

### Effects of altered precipitation

Our findings provide insights into the effects of precipitation changes on Rs in an alpine ecosystem. We found that soil respiration in an alpine steppe exhibited a drastic response to reduced precipitation and a mild response to increased precipitation. Previous studies have suggested that the direction of the Rs response was likely influenced by the water status of an ecosystem with either reductions or increases in precipitation^54^. Our results supported the hypothesis that altered precipitation would change Rs^29^, but differential responses were found for different treatments. In addition, the carbon cycles in arid and semi-arid regions are more sensitive to changes in precipitation^55,56^. In an alpine steppe ecosystem that lacks precipitation, precipitation becomes more important. However, in this study, interestingly, Rs in an alpine steppe ecosystem exhibited a drastic response to precipitation decreases in the Qinghai-Tibetan Plateau.

With the increasing frequency of extreme climate conditions, the mechanism by which drought affects terrestrial carbon cycling has received great attention^57^. Compared with the results of several previous studies in other arid ecosystems^58,59^, reducing precipitation significantly decreased Rs and its component (Ra and Rh) in an alpine steppes (Fig. 4c,d) in our study. Considering the close relationship between Ra and plant photosynthetic activity^60^, the reduced Ra could be attributed to the inhibition of plant growth, which may supply less C substrate for root/rhizospheric respiration^61,62^. Similar to N addition, a larger root biomass means a greater root surface area for respiration^10,22^. Moreover, soil moisture impacts the sensitivity of plant growth, which reduces Ra in this region. The response to soil moisture suggests that decreased precipitation can largely restrict soil respiration and its components, which is more significant if the infrequency of precipitation and number of drought events due to climatic changes were increased^63–65^.

Compared with Ra, decreased precipitation markedly affected Rh, with cumulative Rh reduced in drought conditions. The reduction in Rh could be partly due to the reduction in microbial biomass. A previous study found that decreasing precipitation significantly reduced microbial biomass in a similar grassland community^66^. In addition, reduced precipitation treatment strongly decreased Rh, and drought significantly suppressed Rh. The results conducted in a pine forest are consistent with those of a girdling experiment^67^. Other studies have found that Rs (including Rh) is sensitive to soil moisture in old field systems^68^. Soil water content can change Rh by altering the activity of decomposer microbes and substrate availability^69^. Decreases in microbial biomass and activities under conditions of reduced water availability have been reported in different ecosystems^70^. Therefore, we concluded that the reduced precipitation treatment constrained microbial activity and plant growth, subsequently reducing both Ra and Rh.

Compared with reduced precipitation, the response of Rs and its component to increasing precipitation is not significant (Table 2, Fig. 4e,f). Then, an interesting question arises. Why is Rs not altered under conditions of increased precipitation? We explored several potential reasons that could explain the lack of response of Rs to increased precipitation. First, our study only reported the responses of Rs to short-term increased precipitation (three years), but it remains unclear whether long-term precipitation increased would cause some differences. Second, soil respiration is significantly related to AGB in alpine grasslands on the Tibetan Plateau^71^. Thus, the non-significant response of AGB (Fig. 2a) to increased precipitation may also explain the non-significant response of soil respiration to increased precipitation. Third, the precipitation distribution varied significantly during the last two growing seasons (Fig. 1c). In particular, for the peak plant growth in August, the precipitation amount in 2015 (79.1 mm) was significantly reduced compared with that in 2016 (200.2 mm). The response of AGB and soil moisture to increased precipitation depends on the amount of increased precipitation in alpine grasslands on the Tibetan Plateau^72^ given that both AGB and soil moisture can affect soil respiration^73^. Antecedent precipitation and soil moisture conditions before Rs measurements can also affect the response of soil respiration to precipitation, and these factors also might result in non-significant changes in soil respiration to precipitation^74^. Moreover, the non-significant response of Rs to increased precipitation may also be related to the non-significant difference in soil respiration temperature sensitivity between the control and increased precipitation treatments^74^. Moreover, the highly inorganic N that accumulated in 0–10 cm of soil may be toxic to microbial growth and activities^47^, and we found that soil NO_3_^−^N decreased in the wet year (2016). In addition, high soil moisture potentially stimulated root and microbial activities^52^, and additional precipitation could increase Ra due to enhanced plant growth (Fig. 2a,b), thus leading to non-significant changes in Rs during this period. Finally, the positive effect of nitrogen addition on AGB and nitrate nitrogen increases with increasing the nitrogen addition rate^45,75^. Thus, the non-significant response of soil respiration to nitrogen addition may also be related to its relatively low nitrogen addition rate. Based on these points, it is possible that precipitation-associated water increases collectively remain very important in this alpine steppe. Experiments with longer durations are necessary to better understand Rs and Ra/Rh relationships in future studies. Our findings suggest that Rs might be altered based on precipitation changes in alpine steppe ecosystems in the future.

Increasing precipitation could increase N_2_O^76^, N addition could also increase N_2_O^77^. N_2_O emissions from soils are caused principally by microbial nitrification and denitrification by soil water-filled pore space, mineral nitrogen concentration, temperature and precipitation^76^. Moreover, both CO_2_ and N_2_O as two major greenhouse gases could render global warming. Therefore, more attention should be paid to the N_2_O in future studies.

## Conclusions

Overall, both N addition and altered precipitation did not significantly influence Rs in the first year, but reduced precipitation drastically repressed Rs during the subsequent years. Furthermore, we found that the addition of N significantly increased Ra but had no obvious effects on Rh during the last two years. In addition, altered precipitation significantly influenced Rh but minimally affected Ra. Our results revealed the distinct effects of the addition of N and altered precipitation regimes on heterotrophic and autotrophic respiration. Soil respiration is regulated by soil moisture rather than soil temperature in the Tibetan alpine steppe. These findings suggest that the response of Rs to climate change may be more complex, and our ability to predict the C-N-precipitation interactions will become more challenging in Tibetan alpine steppe ecosystems. In addition, the changes in Rs and its components in response to N addition and precipitation changes merit further attention in future experimental and modelling analyses.

## Methods

### Study site

The study was conducted on the Sanjiaocheng Sheep Breeding Farm (37°18′N, 100°15′E) located in Qinghai province, China. The elevation is 3,286 m. The long-term mean annual precipitation is 387 mm, and the mean annual temperature is 0.08 °C (1980–2012). The soil is classified as chestnut soil^78^ with 61.0% sand, 33.4% silt, and 4.9% clay. The 0- to 30-cm soil properties are as follows: pH 9.5, total organic carbon 20.5 g kg^−1^, total nitrogen 2.5 g kg^−1^ and total phosphorous 0.6 g kg^−1^.

The plant community is dominated by *Stipapurpurea*, *Poa crymophila*, *Artemisia scoparia*, and *Carexivanovae*at at the experimental site. Abundant species include *Heteropappusaltaicus*, *Koeleriaglauca*, *Agropyroncristatum*, *Leymussecalinus*, *Dracocephalumheterophyllum*, *Taraxacummongolicum*, *Cirsiumjaponicum*, *Potentilla multifida*, and *Pedicularis alaschanica*.

### Experimental design

The experimental area was fenced to prevent grazing disturbances, and the experiment was started in May 2013. Thirty plots of 3.3 × 2.7 m^2^ were randomly assigned to 6 treatments within 5 blocks using a randomized block design. Each plot was cut 15 cm around the border with iron sheets (3.0 × 2.4 m). To prevent surface runoff, we inserted an algam into soil at a depth of 20 cm in each plot. Blocks and plots were separated by a 2-m buffer zone. Each plot was split into 3 subplots (subplot 1 for measuring plant biomass and soil characteristics, subplot 2 for measuring carbon fluxes and subplot 3for observing the phenology).

For N treatments, NH_4_NO_3_ (10 g N m^−2^ y^−1^) was evenly sprayed using a sprayer with 1 litre of water into the plots on June and July 15th during each year from 2013 to 2016, and the same amount of water (1 L) was evenly distributed in the control treatments. Previous studies using similar methods to add N indicated that the saturated N load was 8 g m^−2^ year^−1^ at same region in a Tibetan alpine steppe^79^, 8–10 g m^−2^ year^−1^ in temperate grasslands in Inner Mongolia^80^. Thus, the current N addition should be adequate to simulate N saturation in our study site. Precipitation treatments were performed using a steel structure bracket and sunlight-penetrable concave polyvinyl chloride (PVC) boards (1 mm). The steel structure is inclined (north 2.1 m and south 1.4 m), and five PVC boards (each PVC is 24 cm × 352 cm) were alternately arranged with five equal and empty areas, which occupied 50% of the total area. Compared with 50% precipitation decreased treatment, both the contrast treatment and 50% precipitation increased treatment are cut open at the bottom of the PVC boards. Rainwater evenly distributed by a sprayer into the 50% precipitation increased treatment which collected in 50% precipitation decreased treatment through PVC pipes. In addition, previous studies also used ±50% precipitation changes in same region in a Tibetan alpine steppe^81^. Six treatments were included in this study: 50% precipitation reduction treatment (N1P1), natural precipitation (N1P2), 50% additional precipitation treatment (N1P3), 50% reduced precipitation with N addition treatment (N2P1), natural precipitation without N addition (N2P2), and 50% additional precipitation with N addition (N2P3).The six treatments are described in Supplement Table 1.

### Soil respiration measurements

In June 2013, two polyvinyl chloride collars (20 cm in diameter) were inserted into the soil at least 30 cm away from the edge. In each plot, the Rs collar was inserted into the soil to a depth of 3 cm, and the Rh collar was inserted into the soil at least 50 cm. Before measurements, we removed living plants inside the collars once a week. During the growing season (May-October), Rs and Rh were measured with a portable soil CO_2_ flux system (Li-8100, Li-COR, Inc., Lincoln, NE, USA) each month between 9:00 and 12:00 am from 2014 to 2016. Ra was calculated as Rs minus Rh^6^. During the CO_2_ measurements, the soil temperature and volumetric moisture at a 0–10 soil depth were monitored adjacent to the collar using a digital thermometer and a portable TDR-100 soil moisture probe (Spectrum Technologies, Inc., Plainfield, IL, USA), respectively. In addition, soil temperature and moisture were continuously recorded using an EM-50 (Decagon, USA) at a depth of 5 cm every 15 min in each plot.

### Above- and belowground sampling

In this study, aboveground and belowground biomasses were collected during peak growth in the middle of August in 2016. We randomly selected three 0.25 m × 0.25 m quadrants and harvested each quadrant in subplot 1. All plant samples were oven-dried at 65 °C to a constant mass and then weighed to estimate aboveground biomass (AGB).

After AGB sampling, three root augers (4 cm in diameter) were collected in the same quadrants to a depth of 30 cm (10 cm intervals). Then, three cores were mixed as one sample and soaked in water using a 0.5-mm sieve to remove the soil. After BGB presented, root mass was measured and oven dried at 65 °C.

### Soil sampling and inorganic N analyses

Soil samples were collected in the middle of August in each experimental year. Within each plot, soil cores (3 cm in diameter) were collected from the surface layer (0–10 cm) and combined to yield one composite sample. Soil samples were passed through a 2-mm screen in the field after the organic debris and roots were removed, stored in a cooler and transported to the laboratory for the following analyses. The soil’s inorganic N (NO_3_^−^-N and NH_4_^+^-N) was extracted with a 2 M KCl solution and analysed using a AQ1 Discrete Multi-Chemistry Analyzer (SEAL Analytical, USA).

### Statistical analysis

In this study, three-way ANOVA was used to compare the treatment differences based on year, N, precipitation, total NH_4_^+^-N and NO_3_-N. Repeated measures analysis of variance (ANOVA) was used to examine the effects of sampling date, precipitation and non-soil temperature, soil moisture, Rs and its components (Ra and Rh) for the three years as well as their contribution to total Rs (Ra/Rs, Rh/Rs) in 2015 and 2016. All statistical analyses were conducted using SPSS 16.0 (IBM SPSS, Inc., Chicago, Illinois, USA). Unless otherwise stated, the differences were considered statistically significant at *P* < 0.05.

## Electronic supplementary material

Supplement Table1
