# Supplementary material for: Differential responses of heterotrophic and autotrophic respiration to nitrogen addition and precipitation changes in a Tibetan alpine steppe
Source: Sci Rep. 2018 Nov 8;8:16546. doi: 10.1038/s41598-018-34969-5 (PMC6224420; doi:10.1038/s41598-018-34969-5)
Supplement: Supplementary file 1 — Supplement Table1 [file 41598_2018_34969_MOESM1_ESM.docx]

**Differential responses of heterotrophic and autotrophic respiration to nitrogen addition and precipitation changes in a Tibetan alpine steppe**

**Changbin Li^1,2,4^, Yunfeng Peng^3^, Xiuqing Nie^1,2,4^, Yuanhe Yang^3^,Lucun Yang^1,2^, Fei Li^3,4^, Kai Fang^3,4^, Yuanming Xiao^1,2,4^, Guoying Zhou^1,2*^**

1 Key Laboratory of Tibetan Medicine Research, Northwest Institute of Plateau Biology, Chinese Academy of Science, Xining 810008, China; 2 Qinghai Key Laboratory of Qing-Tibet Biological Resources, Xining 810008, China; 3 State Key Laboratory of Vegetation and Environmental Change, Institute of Botany, Chinese Academy of Sciences, Beijing 100093, China; 4 University of Chinese Academy of Science, Beijing 100049, China. *Corresponding author: Northwest Institute of Plateau Biology, Chinese Academy of Science, No. 23 Xinning Rd, Xining 810008, China. Email address: zhougy@nwipb.cas.cn. (G. Zhou), Tel: +86-971-6159630

**Supplementary Material**

**Supplement Table1.** Precipitation change and nitrogen addition in each block(3 m ×2.4 m).

| Treatment | Precipitation change | Nitrogen addition |
| --- | --- | --- |
| 1，N1P1 | 50% Precipitation reduction | 0 g |
| 2，N1P2 | CK | 0 g |
| 3，N1P3 | 50% Precipitation addition | 0 g |
| 4，N2P1 | 50% Precipitation reduction | 205.7 g |
| 5，N2P2 | CK | 205.7 g |
| 6，N2P3 | 50% Precipitation addition | 205.7 g |
